# The Value of Merging Medical Data from Ambulance Services and General Practice Cooperatives Using Triple Aim Outcomes

**DOI:** 10.5334/ijic.5711

**Published:** 2021-10-28

**Authors:** Rosa Naomi Minderhout, Hedwig M. M. Vos, Pierre M. van Grunsven, Isabel de la Torre y Rivas, Sevde Alkir-Yurt, Mattijs E. Numans, Marc A. Bruijnzeels

**Affiliations:** 1 Department of Public Health and Primary Care/LUMC-Campus The Hague, Leiden University Medical Centre, The Hague, NL; 2 Ambulance service Gelderland-South, NL

**Keywords:** Merging medical data, Triple Aim, crowding, ambulance service, General Practice Cooperative

## Abstract

**Background:**

Acute care services are currently overstretched in many high income countries. Overcrowding also plays a major role in acute care in the Netherlands. In a region of the Netherlands, the general practice cooperative (GPC) and ambulance service have begun to integrate their care, and the rapid and complete transfer of information between these two care organisations is now the basis for delivering appropriate care. The primary aim of this mixed-methods study is to evaluate the Netherlands Triage System (NTS) merger project and answering the question: What is the added value of implementing a digital NTS merger in terms of healthcare use and healthcare costs? A secondary question is: What are the experiences of patients and care professionals in different acute healthcare organisations following implementation of the digital NTS merger?

**Methods:**

Patients who made an acute care request during the 12 months before the NTS merge intervention (control period) were compared with matched patients in the 12 months following the start of the NTS merge. Outcomes included difference in healthcare use 30 days after an acute event and patient’ and care professional’ experiences during the intervention period. To assess healthcare costs, we used reference prices updated to 2021.

**Results:**

Compared to patients in the control period, patients in the intervention period were hospitalized less often (52.9% vs 64.4%, p = 0.061) and had fewer emergency department (ED) visits (58.7% vs 69.3%, p = 0.074) in the 30 days following the acute care request. The ED costs were significantly lower during the intervention period compared to the control period (p = 0.042). Furthermore, patients in the intervention period were very satisfied overall with the acute care network (4.63 of 5) and care professionals were fairly satisfied with the cooperation to date (2.73 of 4).

**Conclusion:**

The Triple Aim for acute care can be met using relatively simple interventions, but medical data merging is a prerequisite for achieving more robust results covering on the various aspects of the Triple Aim. These successes should be communicated so that a common language can be developed that will support the successful further implementation of larger scale initiatives.

## Background

Acute care services are currently overstretched in many high income countries, due to a growing demand of patients [1,2,3]. This leads to negative consequences, including temporary limitations to accessibility, a reduced quality of care, and an increased workload for care professionals, that might be avoided [1,2,3,4,5,6].

Overcrowding also plays a major role in acute care in the Netherlands, as illustrated by the 14.7% increase in ambulance deployments between 2013 and 2016. [7] Acute care services across the Netherlands involve many different organisations, including Emergency Departments (EDs), General Practice Cooperatives (GPCs) and ambulance services. The General Practitioner (GP) acts as a gatekeeper at the primary care level, deciding whether to refer a patient to secondary healthcare, resulting in lower healthcare costs for the society as a whole [8]. With a referral from their GP, patients are able to utilize secondary healthcare and are eligible for reimbursement [9]. Patients with medical problems typically visit their own GP during office hours, even when problems are perceived as urgent or threatening [10]. After-hours patients with an acute care request can report to an GPC. In case of a request considered urgent, they can self-refer directly to the ED at all hours, or be transported to the ED by ambulance following a GP visit or as a result of calling the national emergency telephone number 112 [11]. After receiving assistance at an ED, a patient can be hospitalised, referred to a nursing home, receive care at home if necessary, or be referred back home without home-care [12]. These multiple entrance and exit routes increase the pressure on all acute care services [13,14] and the large number of acute care services leads to fragmentation of care. Fragmentation seems associated with increased costs of care, a lower chance of being subjected to clinical best practice care, and higher rates of preventable (re-) hospitalizations [15]. In order to improve the coordination and efficiency of acute care services in the Netherlands and to maintain accessibility in the future, promoting the multilevel integration of care professionals and organisations is critical [16].

In the region of Nijmegen, a city in the South-East of the Netherlands with a population of around 170.000 people, the GPC Nijmegen and ambulance service Gelderland-South started to integrate their care through a rapid and complete transfer of information between these two care organisations [17]. Previously, the telephone was used by the GPC to convey patient information to the ambulance service, resulting in unnecessary delays and loss of information. In 2012 both services installed the validated Netherlands Triage System (NTS) [18]. This sophisticated software is now used by emergency call handlers at both services to prioritise care requests by urgency, and in cases of high urgency, patients who call the GPC can be referred directly to the ambulance service. A digital NTS merger took place in the region in October 2017 and today the appropriate transfer of patient information and cooperation of the organisations are important in supporting referral. Now when a patient with an acute care need calls the GPC Nijmegen and the triage outcome indicates that an ambulance is required, the so-called ‘digital NTS merger’ ensures that a digital report of findings and previous history is sent to the ambulance service Gelderland-South via a secure e-mail service.

This study focused on the evaluation of the digital NTS merger that was introduced in the autumn of 2017 in Gelderland-South. There are several general conceptual evaluation frameworks that focus on the integration of care services, but these do not exclusively emphasise acute care services. The Triple Aim approach, first described by Berwick, Nolan, and Whittington in 2008, uses a multi-stakeholder perspective and focuses on more than just clinical or organizational outcomes [19]. Triple Aim defines improvement of a healthcare system as the simultaneous pursuit of three linked aims: improving the individual experience of care, improving the health of populations, and reducing the per capita cost of healthcare [19,20]. Based on the Triple Aim approach, a framework for evaluation of transitions in acute care services was developed by us, which we used for the current evaluation [21]. The framework we made, is based on a broad view of health rather than focusing on a specific illness. It distinctly explains every step of the evaluation process and can be applied to a heterogeneous group of patients. Our hypothesis was that the digital NTS merger may have yielded “Triple Aim” outcomes, for this context translated to better acute care experiences for patients, reduced unnecessary healthcare use and costs, and the mutual cooperation of and a better work experience for the individual care professionals involved.

The aim of this mixed-methods study is to answer two questions: (1) What is the added value of implementing a digital NTS merger in terms of healthcare use and healthcare costs? (2) What are the experience of patients and care professionals in different acute healthcare organisations after implementation of the digital NTS merger?

## Methods

A commonly accepted point of departure of the Triple Aim approach is to begin with defining a specific population with a high risk of adverse outcomes in healthcare, or a situation needing resolution (a so called ‘’burning platform’’ in healthcare) [22]. We operationalized this by including the most vulnerable in the study, selected by identifying those with potentially the highest risk of adverse outcomes [20], that are related to poor information exchange between GPC and ambulance service [23,24,25]. Aging people, and the increasing proportion of community-dwelling patients with chronic conditions, more frequently use acute care services and require more hospital days than younger people [23,24,26]. The inclusion criteria in this study were:

- Patients who have made an urgent care request to the GPC Nijmegen and according to the triage outcome received ambulance assistance
- Community-dwelling older persons aged over 70 years
- Potentially complex patients identified with multimorbidity (defined as: co-existence of two or more chronic conditions) [25]

Patients who met the inclusion criteria in the 12-month periods preceding and following the data merger (November 2016-November 2017 and November 2017-November 2018) were selected from the practice lists of the ambulance service and the GPC. The follow-up period for each patient was 30 days after the acute event. To determine the experiences of healthcare professionals, we recruited a network of healthcare professionals including emergency call handlers at the GPC Nijmegen and the dispatch centre, together with the ambulance personnel of ambulance service Gelderland-South.

### Primary outcome: healthcare use and cost

Using a before-after design, the primary outcome was the difference in healthcare use in the follow-up period between patients in the control versus the intervention period. Healthcare use was assessed as the number of hospital admissions, admissions to a nursing home, and patient contact with their own GP, with the GPC, or with the ambulance service, all included in a case record form made by the researchers. Case record forms were sent to all GPs for each individual patient selected from the practice lists with are quest to check the electronic medical record and to provide how often a patient used healthcare in the 30 days following an acute request. In anticipation of a poor response rate, researchers IT and SA offered assistance in the form of visits to the GP practices to gather data. To define healthcare costs we used reference prices from the Dutch manual for costing studies [27,28] (2021 edition) [29]. Hospital admission per day was costed at €523.60, an ED visit at €284.90, an emergency ambulance journey at €674.30, an GPC Nijmegen visit at €128.21 [30] and an own GP visit at €10.51 [31]. Other outcomes such as nursing home admission could not be expressed monetarily due to lack of specific data.

### Secondary outcome: the experiences of patients and health care professionals

To address our secondary research question (‘’What are the experiences of patients and care professionals in different acute healthcare organisations after implementation of the digital NTS merger?’’) a cross-sectional questionnaire survey was conducted amongst patients and healthcare professionals following the intervention period. The experiences of professionals were further elaborated through structured discussion in a focus group. As no validated questionnaire exists that can measure patient experiences across the entire acute care network, we developed a questionnaire based on three validated Dutch Consumer Quality Index (CQI) questionnaires (CQI Emergency department, CQI ambulance care and CQI GPC), see appendix A [32,33]. Patient experiences were assessed using several components, including overall satisfaction (experience of accessibility, contact with assistants, confidence in care expertise, expectations, communication, cooperation) and the grading of organisations on a scale of 0–10. Overall satisfaction was measured using the summed mean scores of all components, with a score of 1 indicating very dissatisfied, 3 neutral, and 5 very satisfied. The questionnaire to measure care professionals’ experiences consisted of the validated ‘Leiden Quality of Work Life Questionnaire’, further supplemented with project-specific questions [34]. Questionnaires were sent digitally to the care professionals (see Appendix B). Professional experience was assessed using various subscales such as satisfaction, collaboration with chain partners, collaboration of care professionals within the organisation, completeness of transfer and confidence in the future. A subscale score was the sum of the item score (1 to 4). A focus group was organized to allow in depth discussion and exploration of the cooperation topics and to give professionals the opportunity to provide advice for further improvement. The focus group consisted of two call handlers from the GPC, two ambulance service call handlers, and one ambulance nurse, and took place on June 11^th^ 2019 at the headquarters of the ambulance service Gelderland-South, with IT and SA acting as moderators.

### Statistical analysis

Chi-square tests were performed to determine differences in proportions of binary healthcare use variables (such as hospitalisation in the 30 days after the acute care request [yes or no], ED visit, contact with ambulance service) between the control and intervention period. T-tests were used to determine differences in total healthcare costs per individual. Estimated healthcare costs were individually assessed by multiplying average cost price by healthcare use. Descriptive statistics were used to evaluate patient’ experiences. We assessed the differences between subscale scores for the different professions by comparing the mean scores using one-way ANOVA tests. The effect of sex and average working hours per week between different professions was corrected for using linear regression. The audio recording of the focus group was transcribed verbatim, coded and labelled by IT and SA, and checked by RNM.

Statistical analyses were performed using SPSS Statistics 24 software program (IBM, Armonk, NY, USA). The study was registered and approved by the medical research ethics committee of Leiden University Medical Centre (LUMC), P18.167.

## Results

### Primary outcome: healthcare use and costs

746 patients during the control period and 423 patients during the intervention period were included from the practice lists of the ambulance service and the GPC. We were able to complete file research on 163 of patients in the control period and on 104 patients in the intervention period. The reduced number of patients in the study population was due mainly to the difficulty of data collection in GP practices. Many GPs, burdened by their high workload, were hesitant to cooperate with the study by checking the electronic medical record and filling in how often a patient used healthcare in the 30 days following an acute request. Patient age and gender did not differ significantly between the two groups (p = 0.338, p = 0.328 respectively). Compared to patients in the control period, patients in the intervention period were hospitalized less often (52.9% vs 64.4%, p = 0.061) and had fewer ED visits (58.7% vs 69.3%, p = 0.074) in the 30 days after the acute care request. The number of nursing home admissions was lower during the intervention period compared to patients in the control period (2.9% vs 14.8%, p = 0.002), and fewer ED costs were incurred compared to the control period (p = 0.042). However, the opposite trend was seen for the GPC (p = 0.002). All results are shown in ***Table 1***.

**Table 1:** Healthcare use and estimated costs (in 2021€).


TOTAL GROUP (N = 267)	CONTROL PERIOD (N = 163)	INTERVENTIONPERIOD (N = 104)	P-VALUE

**Patients characteristics**			

Age, mean (±SD), years	79.78 (6.6)	78.95 (6.2)	0.338

Sex, n male (%)	66 (40.7)	46 (46.9)	0.328

**Healthcare use in the 30 days after acute request**			

Hospitalisation, n yes %	64,4	52.9	0.061

ED, n yes (%)	69.3	58.7	0.074

GPC, n yes %	11.8	16.3	0.292

Emergency ambulance ride, n yes %	5.0	3.8	0.660

Own GP, n yes %	78.3	84.6	0.200

Admission to a nursing home, n yes %	14.8	2.9	0.002

Need of a district nurse, n yes %	22.1	23.4	0.848

**Number of times healthcare use, when answer was yes**			

Number of hospital admission, mean (±SD)	1.15 (0.4)	1.15 (0.4)	0.933

Number of hospitalization days, mean (±SD)	4.10 (7.4)	3.37 (5.7)	0.402

Number of ED visits, mean (±SD)	1.09 (0.3)	1.11 (0.3)	0.603

Number of GPC visits, mean (±SD)	1.26 (0.7)	1.81 (1.47)	0.161

Number of own GP visits, mean (±SD)	2.75 (1.9)	2.64 (1.8)	0.684

Number of emergency ambulance ride, mean (±SD)	1.13 (0.4)	1.25 (0.5)	0.624

**Average costs per patient calculated with the reference prices [27,28,29,30,31]**			

Hospitalization costs, mean (±SD)	€2147 (3851.5)	€1764 (2969.8)	0.625

ED costs, mean (±SD)	€215 (161.7)	€186 (172.0)	0.042

Emergency ambulance ride costs, mean (±SD)	€38 (173.2)	€32 (172.7)	0.627

GPC costs, mean (±SD)	€19 (61.2)	€36 (111.4)	0.002

Visit own GP costs, mean (±SD)	€22 (21.5)	€23 (20.5)	0.516

Costs together, mean (±SD)	€2468 (3959.6)	€2046 (3060.7)	0.671


### Secondary outcome: patient and healthcare professional experiences

#### Patients experiences

We found 40 patients of 104 patients (38%) in the quantitative section to be available for questionnaire research regarding the intervention period due to drop-out for various reasons, with the most common reason being deceased. Of those completing the questionnaire, overall satisfaction with acute care was very high (4.63 [0.4 SD] out of 5). Acute services also received very high scores (out of 10), with 7.9 (1.2 SD) for GPC call handlers, 7.98 (1.2 SD) for the overall GPC organisation and 8.67 (1.0 SD) for the ambulance nurses (***Table 2***). Nevertheless, 15% of the patients were not connected to a call handler within the prescribed two minutes. The final questionnaire question was: ‘If you could name one thing, what would you like to change?’ Of those who answered this open question, a faster transfer, better cooperation between the various care professionals, and less waiting at the ED were mentioned.

**Table 2:** Patients experiences with acute care.


	TOTAL GROUP (N = 40)

**Patients characteristics**	

Age, mean (±SD), years	78.72 (4.5)

Sex, n male (%)	20 (50.0)

Hospitalisation, n yes (%)	16 (40.0)

ED visit, n yes (%)	20 (50.0)

Contact GPC, n yes (%)	4 (10.0)

Contact ambulance service, n yes (%)	3 (7.5)

**Satisfaction, 1 means very dissatisfied, 5 very satisfied**	

Overall satisfaction (±SD)	4.63 (0.4)

**Average score, 0 means bad and 10 means excellent**	

Call handlers GPC (±SD)	7.9 (1.2)

GPC organisation (±SD)	7.98 (1.2)

Ambulance nurses (±SD)	8.67 (1.0)


#### Experience of care professionals

Of the 160 CAWI questionnaires sent to care professionals, 76 (48%) responded: 21 GPC call handlers, 13 dispatch centre call handlers and 42 ambulance nurses. From a maximum score of 4 (completely satisfied), the average score for the total group of 76 care professionals was 2.73(±0.5 SD). The GPC call handlers scored significantly higher on all topics compared to the dispatch centre call handlers and the ambulance nurses (3.15 vs. 2.73 and 2.52, p < 0.01 for both), ***Table 3***. Correction for sex (β = 0.134 p = 0.186) and average working hours per week (β = –0.008, p = 0.302) using linear regression did not negate this effect.

**Table 3:** Experience of care professionals, CAWI questionnaire.


	TOTAL GROUP (N = 76)	CALL HANDLER GPC (N = 21)	CALL HANDLER DISPATCH CENTER (N = 13)	AMBULANCE NURSE (N = 42)	P-VALUE

**Care professional characteristics**					

Age, mean (±SD), years	46 (9)	45 (10)	50 (11)	46 (9)	0.400

Sex, male : female, n	30 : 46	0 : 21	6 : 7	24 : 18	<0.01

Average working hours a week, mean (±SD)	29.17 (7.4)	21.19 (7.0)	30.31 (5.5)	32.81 (4.7)	<0.01

Work experience in profession, mean (±SD), years	14.76 (9.7)	14.71 (8.9)	14.54 (9.5)	14.86 (9.7)	0.994

Work experience within organisation, mean (±SD), years	10.87 (7.3)	9.95 (5.8)	13.38 (8.7)	10.55 (7.5)	0.383

**Number of points per topics, maximum score 4**					

Satisfaction, mean (±SD)	2.73 (0.6)	3.35 (0.3)	2.77 (0.5)	2.41 (0.6)	<0.01

Collaboration chain partners, mean (±SD)	2.70 (0.4)	2.90 (0.3)	2.65 (0.4)	2.61 (0.4)	0.017

Collaboration own organisation, mean (±SD)	3.06 (0.3)	3.34 (0.3)	2.91(0.3)	2.95 (0.3)	<0.01

Completeness transfer, mean (±SD) Confidence in future, mean (±SD)	2.44 (0.6) 2.72 (0.8)	2.85 (0.6) 3.29 (0.6)	2.62 (0.4) 2.69 (0.6)	2.18 (0.6) 2.45 (0.8)	<0.01<0.01

All above components together, mean (±SD)	2.73 (0.5)	3.15 (0.3)	2.73 (0.5)	2.52 (0.4)	<0.01


A focus group was organised to discuss and explore cooperation topics in more detail and to allow professionals the opportunity to provide advice for further improvement. Various quotes (Q) from focus group members can be found in appendix C. All partners confirmed that the digital NTS merger allows faster transfer from the GPC to the ambulance service, and that less discussion is required prior to referral of a patient between organisations as transfer is easier to arrange (Q1). Further, care professionals reported less dissatisfaction among patients because they no longer had to repeatedly relate their case details to every individual care provider. As an example, an ambulance nurse can access patient details received by the call handler per telephone (Q2). However, ambulance nurses were still not completely satisfied with the content of the merged digital NTS, as they reported a large discrepancy between the information received from dispatch centre call handlers and the GPC call handlers (Q3, Q4). The extensive transfer details received from the GPC call handlers were not always felt to be useful or complete. This problem was partly caused by GPC call handlers not always being aware that some information available on their own computer screen, such as the medication list and patient history, was not automatically sent to the ambulance service (Q6–8). While the digital NTS merger was a worth while attempt to improve patient information transfer, there is clearly still room for improvement. During the discussions it quickly became apparent that the partners understood little about each other’s organisation and work. As an example, they were unaware that they used the same NTS triage system, did not know how many ambulances were present in the region, where the chain partners worked, and so on (Q9–10). Nevertheless, the chain partners were in favour of further improvement of their collaboration, and mentioned during the focus group that this was the first opportunity to meet (Q11–12). They also indicated a preference for clear agreements concerning the information included in the transfer details and a clear agreement between the organisations on the determination of urgency. Joint trainers and courses were suggested (Q12–13) and in the future all chain partners would prefer to work together under one roof (Q14).

## Discussion

The purpose of this evaluation was to determine the added value of implementing a merged digital NTS for acute care users with the highest risk for adverse outcomes. The Triple Aim seems to have been achieved in this population via this intervention.

Regarding the population health aspects of the Triple Aim, which in this context is healthcare use in a specialist setting, we noticed that the digital NTS merger was possibly beneficial with a significant reduction in admission to nursing homes (p = 0.002) and a reduction in hospital admissions and ED visits with a possible trend towards significance (p = 0.065, p = 0.074). As for healthcare costs, we noted a decrease in average costs per patient calculated based on reference prices. During the intervention period, patients visited their own GP more often but the difference was not significant. Patients in the intervention period were also responsible for significantly greater costs at the GPC. A shift from intramural to extramural care may be underway and deserves further investigation. Earlier studies have reported conflicting results regarding the effectiveness of care coordination services, with variation probably attributable to differences in the intensity and duration of services [35]. An evaluation of participation in an ED-initiated community-based program reported significantly fewer ED visits and significantly more primary care visits [36]. Since ED care is more expensive then primary care, it appears that the cost benefits of the program are significant [36,37]. The lack of a post-hoc power analysis does not allow us to address whether our negative conclusions are demonstrative of a lack of association between NTS-patient care and clinical outcomes, or reflective of an underpowered study. Power analysis requires accurate knowledge of outcome standard deviations within the analysis cohort, which was here not available given the novelty of the NTS approach [38].

Besides population health and costs, overall satisfaction of patients with the acute care services was very high. The relatively high drop-out rate in the retrieval of questionnaires among patients during the intervention, made interpretation of qualitative data difficult. However, the results offer a glimpse into patient experiences with acute care in the region. The experience of care professionals also plays an important role and addressing the needs of this group adds a fourth policy aspect, leading to our referencing as ‘Quadruple Aim’ [39]. Regarding current satisfaction, the care professionals were generally fairly satisfied with cooperation to date. However, we noted major differences between the various professions, with the most satisfied group being the GPC call handlers. Focus group comments cast some light on differences in satisfaction, which seemed to be often linked to issues such as a lack of understanding of the logistical details of digital transfer. Joint trainers and courses were suggested to improve collaboration, as well as more frequent meetings to gain a better understanding of each other’s work. Other studies have reported less positive results concerning collaboration, but arrived at similar conclusions. Our results are in line with a Norwegian study reporting that smooth cooperation between GPs and ambulance personnel requires that both parties better understand each other’s procedures and roles [40].

Our results provide an early indication of the considerable promise of medical data merging. Given the small numbers in this study and the tentative but not conclusive results, we recommend a replication of this study in a larger context. Other studies have shown that collaboration between GPCs and ambulance services allows patients to avoid transfer to an ED, potentially avoid subsequent hospital admission, reduce cost and improve quality of care for those not actually needing hospital services [41]. However, none of these studies assessed use of services in the days following an acute call, a unique aspect of the current study. Since the design of our study was before-after, without the possibility for patient randomization, we should be careful when inferring causal effects due to the digital NTS merger. Other factors may have also played a role, such as the significantly lower number of nursing home admissions during the intervention period, an outcome that may have been influenced by ‘aging-in-place’ policies in the Netherlands that in recent years have substituted home care for nursing home admissions [42]. We therefore propose a randomized design for a follow-up study. We further learned that patient data collection in acute care is particularly challenging, as the first report is received by the GPC, subsequently forwarded to the ambulance service, and all subsequent treatment by different care providers must eventually be retrieved from the GP’s patient file once completed. As a result, projects of this type have often ‘just’ started, without scientific evaluation. Additionally, due to the high GP practice workloads, GP’s often feel unable to cooperate, an understandable reticence considering the large amount of work required to collect data from the medical records of each individual patients. The initial response rate of GPs via digital channels was small, but subsequent approaches by telephone were considerably more successful. We suggest investing in a research staff member specifically for data analytics and recommend the use of linked datasets between all acute services, understanding these are often not yet available in the Netherlands.

A systematic review of literature on the Triple Aim framework in the context of healthcare concluded that providers generally struggle due to a lack of guidance and an absence of a composite set of measurements that allow performance assessment. Available data therefore often lack clarity regarding the selection and implementation of purposeful measures [43]. We propose that acute care initiatives should be evaluated withing a general framework in a consistent manner, an approach which will promote understanding of existing problems faced during the provision of acute services.

The results of this study suggest that a shift from intramural to extramural care is also possible in the case of acute care and may contribute to the sustainability of our healthcare system: a better quality of care requiring fewer resources, and acute care in the right place at the right time.

## Conclusions

The Triple Aim for acute care can be met using relatively simple interventions, but medical data mergingis a prerequisite for achieving more robust results covering on the various aspects of the Triple Aim. These successes should be communicated so that a common language can be developed that will support the successful further implementation of larger scale initiatives. The final aim of all initiatives should be an optimal acute care network for all citizens that is demonstrated by solid research.

## Additional File

The additional file for this article can be found as follows:

10.5334/ijic.5711.s1 Supplementary File 1. Appendix.
