# Supplementary material for: The Value of Merging Medical Data from Ambulance Services and General Practice Cooperatives Using Triple Aim Outcomes
Source: Int J Integr Care. 2021 Oct 28;21(4):4. doi: 10.5334/ijic.5711 (PMC8555478; doi:10.5334/ijic.5711)
Supplement: Supplementary File 1. — Appendix. [file ijic-21-4-5711-s1.pdf]

Appendix A: Care experience of patients with regard to the digital NTS merge

**Q1**	Did you receive an assistant on the phone within 2 minutes when you called the out-of-ours GP service?
**Q2**	What score do you give the out-of-hours GP service as an organisation? 0 means very bad, 10 means excellent
**Q3**	What score would you give the assistant on the phone of the out-of-hours service? 0 means very bad, 10 means excellent
**Q4**	What did you expect form the assistance when you contact them?
**Q5 A**	The care professionals have met my expectations
**B**	I was happy that the assistant of the out-of-hours GP service deployed an ambulance
**C**	Thanks to the good cooperation between the out-of-hours GP service and ambulance service, I have received good care
**D**	The ambulance nurse was aware of medical information that I had discussed on the phone with the assistant of GP service
**E**	I am satisfied with the care I received from the GP service
**F**	I got the help as soon as I wanted
**G**	I was confident in the expertise of the assistant on the phone
**H**	I think the assistant has made the right choice for me to deploy an ambulance
**I**	I was confident with the treatment
**J**	I got the help I needed
**K**	I am satisfied with the care I received from the ambulance
**L**	If a friend of mine had the same complaints, I would advise him to contact the GP service
**Q7**	Did the ambulance nurse tell you who to contact if you were concerned about your health problem after he left?
**Q8**	What score would you give the ambulance nurse? 0 means very bad, 10 means excellent
**Q9**	I have spoken or seen the following care professionals after ambulance care:
**Q10**	Did the different care professionals (as mentioned in Q10) work together?
**Q11**	Was the following care professional (doctor or nurse) aware of the information that you had discussed with the ambulance
**Q12**	How was your health before the report?
**Q13**	How is your health at the moment?
**Q14**	What would you like to change if you can name one thing?

Appendix B: Computer Assisted Web Interviews (CAWI) for care professionals with regard to the digital NTS merge

Subject	Code	Statement
**Satisfaction**	S1	The collaboration within the project has been of added value for the quality of patient care
	S2	The project has improved the cooperation between the chain partners
	S3	Due to the increasing cooperation with other care providers, I am afraid of losing my autonomy
	S4	The increasing cooperation with other care providers is of added value to me as a care provider
	S5	Due to the increasing cooperation between care providers, I am afraid to give up some of my professionalism
	S6	The digital NTS merge gives me confidence in the future
	S7	The digital NTS merge motivates to further expand the collaboration
**Collaboration of different care professionals with chain partners**	CC1	Clear working agreements have made between chain partners
	CC2	There is a good mutual cooperation with my chain partners
	CC3	I see other care providers within the acute care chain as colleagues rather than as competitors
	CC4	The mutual communication is going well between my chain partners
	CC5	I have faith in the competence of my chain partners
	CC6	I feel appreciated by my chain partners
	CC7	My chain partners criticize my organisation in an annoying way
	CC8	My chain partners offer me a helping hand when needed
	CC9	I experience respect between the various chain partners
	CC10	I feel free to talk to my chain partners about the policies they have implemented
	CC11	The chain partners have a good understanding of everyone’s responsibilities
**Collaboration of care professionals within the**	CO1	Clear work agreements have been made within my organisation
	CO2	There is a good mutual cooperation within my organisation

**own organisation**	CO3	I see other care providers within my organisation as colleagues rather than as competitors
	CO4	The mutual communication is going well between my colleagues
	CO5	I have faith in the competence of my colleagues
	CO6	Within my organisation I feel appreciated by my colleagues
	CO7	Within my organisation, employees criticize each other in an annoying way
	CO8	Within my organisation, my colleagues offer a helping hand when needed
**Completeness transfer**	T1	When I transfer a patient, I receive complete and correct information from my chain partner
	T2	Since the NTS merge, I have received more complete and correct information from my chain partner when transferring a patient than before the project.
	T3	When a patient is transferred, I receive complete and correct information from my colleagues within my organisation
	T4	Since the NTS merge, I have received more complete and correct information from my colleagues within my organisation
**Confidence in the future**	F1	I have faith in good cooperation within the future acute care

The answer categories of all these statements were as follows: (i) Strongly disagree (ii) Disagree, (iii) Agree and (iv) Strongly agree.

**Wat is your profession?**	a) Ambulance nurse<br>b) Ambulance service medical dispatcher<br>c) Out-of-hours GP service medical dispatcher
**Wat is your age?**	.. year
**Wat is your gender?**	a) Male<br>b) Female
**How many hours do you work per week on average?**	.. year
**How many years of work experience do you**	.. year

**have in your profession?**	
**How many years of work experience do you have within your organisation?**	.. year

Appendix C: Quotes from the focus group

Topic	**Quote**
Satisfaction	Q1. Call handler ambulance service: ''Well I think it's positive that we don't have to start a discussion anymore. They no longer have to 'sell' the ambulance ride to us..''
	Q2. Ambulance nurse: '' So there is great benefit in that the patient does not have the dissatisfaction of telling the story twice.''
	Q3. Ambulance nurse: '' We notice that we get a whole piece of text that is written in a completely different way and order than they [call handlers ambulance service] write […] and which I cannot do very much.''
	Q4. Call handler ambulance service: ''Our text is much shorter and more succinctly addressed to an urgency and you [call handlers out-of-hours GP service] are more elaborate. That's the difference I think.''
Completeness transfer	Q5. Ambulance nurse: ''I cannot read much information about the patient, such as medication use and history.''
	Q6. Call handler out-of-hours GP service: ''..because of course we have patients' history and medication visible in our computer system, does that automatically go along? I was curious about that.''
	Q7. Ambulance nurse: ''No, I would really like to have that information…..''
	Q8. Call handler out-of-hours GP service: ''That is why we have not filled it in until now, because we already see this information on our computer screen.''
Collaboration	Q9. Call handler out-of-hours GP service: ''Because you [call handler ambulance service] also do triage via NTS?''
	Q10. Call handler ambulance service: ''Yes I do.''
	Q11. Call handler out-of-hours service: ''… which is why I wanted to participate this focus group. I want to be able to work together more easily.''
	Q12. Call handler ambulance service: ''Sure, I want to work towards better cooperation.''
Future	Q13. Call handler out-of-hours GP service: ''If you take a look behind the scenes of the other organisation, you know exactly what you are talking about.''
	Q14. Call handler ambulance service: ''You work together by being together!''
